# Skin Color Analysis of Various Body Parts (Forearm, Upper Arm, Elbow, Knee, and Shin) and Changes with Age in 53 Korean Women, Considering Intrinsic and Extrinsic Factors

**DOI:** 10.3390/jcm13092500

**Published:** 2024-04-24

**Authors:** Eun Ju Lee, Ja Hyun Ryu, Ji Hwoon Baek, Yong Chool Boo

**Affiliations:** 1 Skin Research Center, Dermapro Ltd., Seoul 06570, Republic of Korea; pololi-3@hanmail.net (E.J.L.); seaskyjh@gmail.com (J.H.R.); 2 Department of Molecular Medicine, School of Medicine, Kyungpook National University, Daegu 41944, Republic of Korea; 3 BK21 Plus KNU Biomedical Convergence Program, Department of Biomedical Science, The Graduate School, Kyungpook National University, Daegu 41944, Republic of Korea; 4 Cell and Matrix Research Institute, Kyungpook National University, Daegu 41944, Republic of Korea

**Keywords:** skin color, elbow, knee, forearm, upper arm, thigh, shin, body color, age, lightness, melanin index, erythema index

## Abstract

**Background/Objectives**: Skin color is innately determined by race and other genetic factors, and it also undergoes acquired changes due to various intrinsic and extrinsic factors. Previous studies on skin color have mainly focused on the face, and research has recently expanded to other body parts. However, there is limited information about the age-dependent changes in the skin color of these body parts. The purpose of this study is to analyze the differences in skin color between various body parts and the changes in skin color of each body part with age. **Methods**: This study examined the skin color of 53 Korean women subjects evenly distributed in age from the 20s to 60s on several body parts: forearm, upper arm, elbow (extended or folded), knee (extended or folded), thigh, and shin. The lightness (L*), redness (a*), and yellowness (b*) were measured using a spectrophotometer, and the individual typology angle (ITA°) was calculated from the L* and b* values. The melanin index and erythema index were measured using the mexameter. **Results**: The results showed that the elbow skin had the lowest L* and ITA° values and the highest a* and b* values among the examined body parts, followed by the knee. The melanin index and erythema index were also high in the skin of these body parts. In the analysis of age-dependent changes in the skin color of various body parts, the forearm skin exhibited the most notable decrease in the L* and ITA° values and increases in the a* and b* values, followed by upper-arm skin. The melanin and erythema indices in the forearm also increased as the subjects aged, whereas those in the elbow and knee rather decreased with age. **Conclusions**: This study suggests that differences in intrinsic and extrinsic skin aging in various body parts may be expressed as different changes in skin color and raises the need for cosmetic and dermatological research to identify the physiological significance of these changes.

## 1. Introduction

Human skin is not only an organ that performs unique physical, physiological, and sensory functions but also an important channel for expressing one’s characteristics and charms [1]. Human skin color varies depending on race, gender, age, and individual [2]. Even within the same person, it varies by body part and changes depending on the region, season, lifestyle, health, and nutritional status [3,4,5]. 

Skin color is largely determined by the type, amount, and distribution of melanin pigment in the epidermis [6]. Melanin is synthesized in melanocytes located at the basal layer of the epidermis and transported to surrounding keratinocytes [7,8]. Melanin synthesis and transport are regulated by multiple cell signaling pathways in response to various internal and external stimuli [9,10]. Abnormally high or low levels of melanin in certain skin areas may cause hyperpigmentation and hypopigmentation disorders, respectively [11,12,13].

Skin color is also significantly influenced by other pigments, such as bilirubin and hemoglobin in the dermis and subcutaneous tissue [14,15]. Hemoglobin in the blood is usually red, but when there is a lack of oxygen, it turns blue. It affects skin color depending on the distribution of capillaries and the degrees of vascular dilatation, blood flow, and oxygen supply [16]. Advanced glycation products produced by oxidative reactions between proteins and carbohydrates also affect skin color [17]. Ultraviolet rays stimulate melanocytes through various mechanisms, promoting the synthesis and distribution of melanin, as well as causing erythema and inflammatory reactions [18,19]. Skin wounds, inflammation, and aging also cause morphological and pathological changes accompanied by skin color changes [12,20,21]. 

In previous studies, skin color research has mainly focused on the face, neck, and hands [22,23,24] but has recently expanded to include other body parts [25,26]. Various body parts have different degrees of exposure to mechanical stress, friction, ultraviolet rays, air pollution, and other factors, so their skin colors can vary [27,28]. Additionally, significant changes in the skin color of each body part can be caused by pregnancy, physical and chemical injuries, and various skin diseases, such as psoriasis, eczema, and acne [12,29,30]. In particular, in the case of elbows and knees, hyperkeratosis, called FAEDS (frictional asymptomatic darkening of the extensor surfaces), may occur [31]. However, there is limited information about the age-dependent changes in skin color of various body parts.

After birth, various body parts experience different conditions coordinated by diverse intrinsic and extrinsic factors, and thus, they undergo adaptation or aging, which involves a change in skin color [3,32]. This hypothesis motivated us to examine the differences in skin color of various body parts and their color changes with age. In the present study, the skin color of 53 Korean women subjects evenly distributed in age from the 20s to 60s was measured on several body parts: forearm, upper arm, elbow (extended or folded position), knee (extended or folded position), and shin. The test was conducted on female subjects because it was considered that women are generally more interested in skincare and are more likely to participate in skin testing. It was also considered that as women go through menopause, their skin would undergo significant changes. The results of this study were discussed in consideration of intrinsic and extrinsic factors associated with skin aging. 

## 2. Materials and Methods

### 2.1. Clinical Study and Human Subjects

A clinical study was conducted to examine skin color on several body parts in human volunteers of different age groups. The test protocol (No. 1-220777-A-N-01-DICN21182) was approved on 9 September 2021, by the Institutional Review Board of Dermapro Ltd. (Seoul, Republic of Korea), and the test proceeded from September to October 2021. This study was conducted in accordance with ethical principles based on the Declaration of Helsinki. Human subjects were selected from healthy volunteers who met both the inclusion and exclusion criteria described in a previous study [23]. In particular, volunteers with skin abnormalities, such as spots, acne, erythema, and capillary dilatation, in the test skin sites were excluded from the selection of the subjects. The purpose and process of the test were explained to the subjects and written consent to participate in the test was obtained from them before the test. A total of 53 Korean women participants aged from 21 to 67 years were included in this study. 

### 2.2. Body Sites for Skin Color Assessment

**Figure 1:**
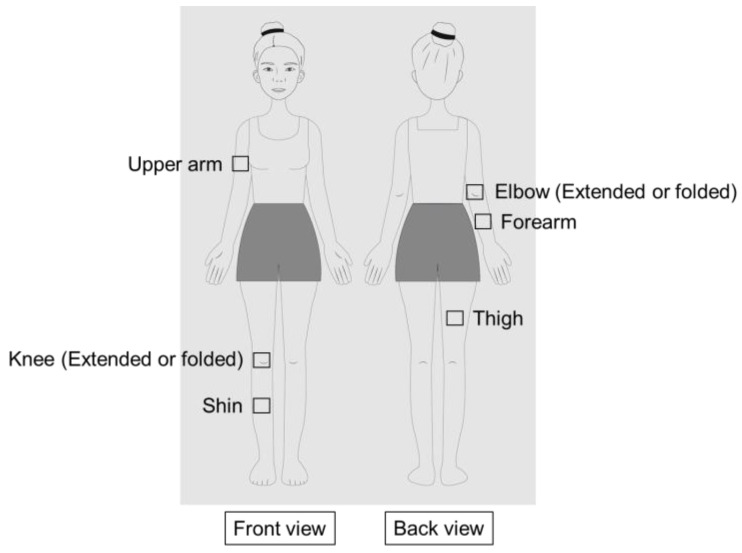
Skin sites for color assessment in various body parts. The skin color of the elbow and knee was measured in their fully extended (180°) or partially folded (110°) position.

The skin color of the right or left body parts was measured depending on the subject’s dominant hand. In the case of ambidextrous subjects, measurements were taken on the right side. Skin color assessment sites (3 cm × 3 cm) were designated on various body parts, such as the right or left upper arm, forearm, elbow, shin, thigh, and knee (Figure 1). Skin color was measured within these sites, avoiding visible wounds, scars, moles, or hairs. In the case of the elbow and knee, the skin color was measured when the elbow and knee were fully extended to 180° (extended elbow and extended knee) or partially folded to 110° (folded elbow and folded knee).

### 2.3. Skin Color Assessment Methods 

The subjects visited the research center on the day of the skin color assessment. They rested for 20−30 min in a laboratory maintained at 22 ± 2 °C and 50 ± 5% relative humidity, followed by skin color assessment. 

Skin color is expressed with the degree of lightness (L*), degree of green to red (a*), and degree of blue to yellow (b*), based on the Commission Internationale de l’Eclairage Lab color space [32]. The, L*, a*, and b* values of the skin sites were measured using a spectrophotometer CM-2500d (Minolta, Tokyo, Japan) [33]. The individual typology angle (ITA°) representing skin color was calculated from the measured L* and b* values using the equation: ITA° = (arc tangent [(L* − 50)/b*])(180/π) [34,35]. The melanin index and erythema index were measured using the mexameter MX18 (Courage + Khazaka electronic GmbH, Cologne, Germany) [36]. The probe of this instrument emits three different wavelengths of light (green, λ = 568 nm; red, λ = 660 nm; and infrared, λ = 880 nm), and its receiver detects the light components reflected by the skin. The measurements were repeated three times and averaged.

### 2.4. Statistical Analysis 

The statistical analysis of data was conducted using the SPSS statistics version 22 software program (IBM, Chicago, IL, USA). Data are expressed as the mean ± standard deviation (SD). The one-way ANOVA and Tukey test were used in the multiple comparisons of data at the *p* < 0.05 level. The Pearson correlation test was used to examine whether and how strongly two variables are linearly associated at the *p* < 0.05 level.

## 3. Results

### 3.1. Information of Human Subjects 

A total of 53 female subjects ranging in age from 21 to 67 participated in the test. As shown in Table 1, the ages of the subjects were evenly distributed in the 20s, 30s, 40s, 50s, and 60s, and the overall average age was 44.72. 

Table 2 shows the subjects’ occupation and life-related information obtained through the questionnaire. The subjects in the 20s age group are mostly students or workers, the subjects in the 30s age group are mostly workers or housewives, and most subjects in the 40s to 60s age groups are housewives. More than 75% of subjects in all age groups responded that they mainly engage in activities indoors. Most subjects sleep normally. Most subjects’ daily exposure time to sunlight or ultraviolet rays is less than 3 h, and only a few respondents said they are exposed to repeated friction. Although smoking status is not an exclusion criterion, there were no smokers among the subjects. The majority of subjects were right-handed, followed by ambidextrous and left-handed subjects.

Table 3 provides the information on the subjects’ skin characteristics, obtained through the questionnaire. It shows the facial skin types of the subjects and the skin moisture, oil, texture, thickness, and pigmentation of their arms and legs. The older the age group, the more subjects responded that they had a dry skin type, reaching 90% in the 60s group. Among the younger subjects, some responded that they had dry-oily or problematic skin types. Many of the subjects responded that their arms and legs lacked moisture, and this was more common in the older groups. There were a significant number of subjects who responded that the skin oiliness in their arms and legs was below average, regardless of the age group. We expected certain changes in body skin texture, thickness, and pigmentation with age, but the responses of the subjects did not fully support or reject this prediction.

### 3.2. Differences in Skin Colors of Various Body Sites 

**Figure 2:**
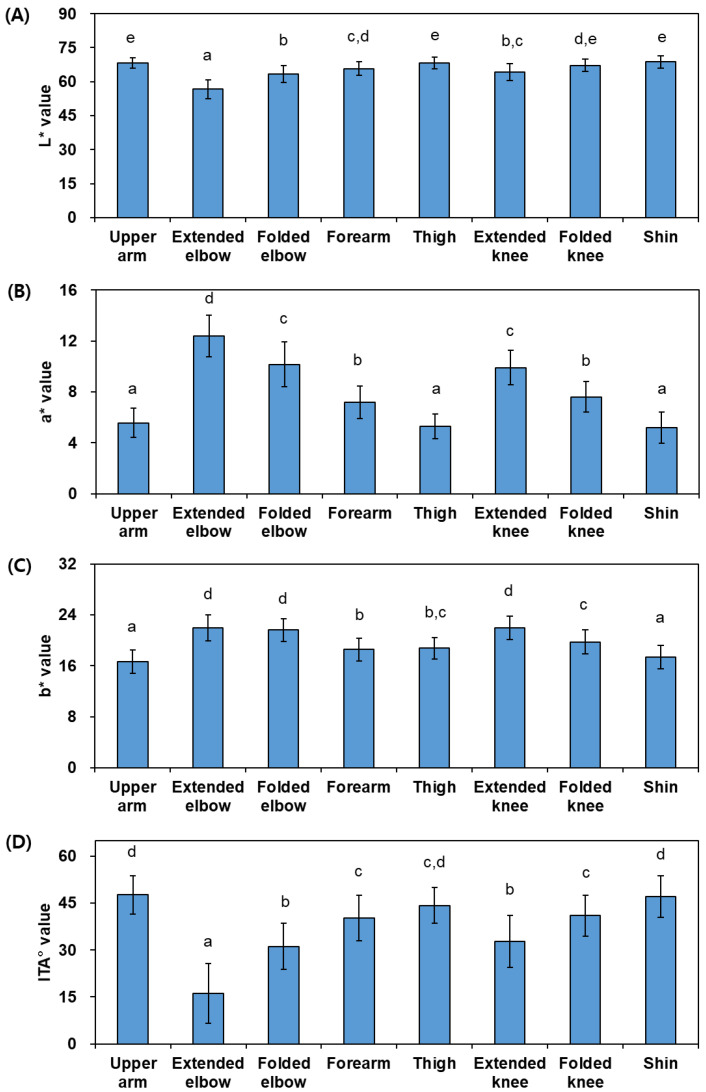
Skin color parameters, such as the L* (**A**), a* (**B**), b* (**C**), and ITA° values (**D**), of various body parts in human subjects (*N* = 53). Bars with different letters have significantly different mean values at *p* < 0.05.

The L*, a*, and b* values measured at skin sites of various body parts using a spectrophotometer are shown in Figure 2A–C. ITA° values calculated from the L* and b* values are shown in Figure 2D. Among the measured skin color parameters (L*, a*, and b* values), the difference in the a* value between body parts was greater than that in the L* or b* value. The variance in the ITA° value between different body parts was as large as that in the a* value. 

The L* values were smaller in the following order: extended elbow < folded elbow ≤ extended knee ≤ forearm ≤ folded knee ≤ upper arm, thigh, shin. The a* values were greater in the following order: extended elbow > folded elbow, extended knee > folded knee, forearm > upper arm, thigh, shin. The b* values were greater in the following order: extended elbow, folded elbow, extended knee > folded knee ≥ thigh ≥ forearm > shin, upper arm. The ITA° values were smaller in the following order: extended elbow < folded elbow, extended knee < forearm, folded knee ≤ thigh ≤ shin, upper arm. 

**Figure 3:**
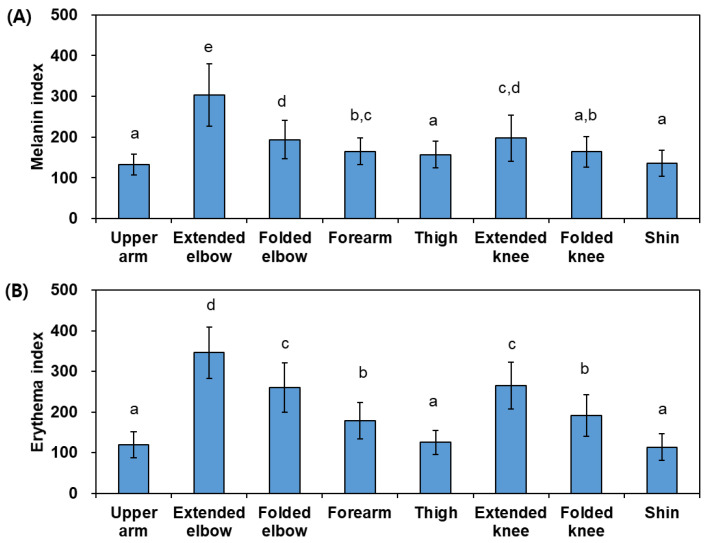
Skin melanin index (**A**) and erythema index (**B**) of various body parts in human subjects (*N* = 53). Bars with different letters have significantly different mean values at *p* < 0.05.

The melanin index and erythema index measured at skin sites in various body parts using the mexameter are shown in Figure 3A,B. Melanin indices were greater in the following order: extended elbow > extended knee, folded elbow ≥ forearm ≥ folded knee ≥ thigh, upper arm, shin. Erythema indices were greater in the following order: extended elbow > folded elbow, extended knee > folded knee, forearm > thigh, upper arm, shin. 

These results indicate that the skin color of the elbow and knee is darker and redder than that of the upper arm, thigh, and shin, and the skin color of the forearm is of medium brightness and color. It is also indicated that the skin color of the elbows and knees in an extended position is darker and redder than in a folded position, and in the same extended position, the skin color of the elbows is darker and redder than that of the knees.

### 3.3. Age-Dependent Changes of Skin Colors of Various Body Sites 

**Figure 4:**
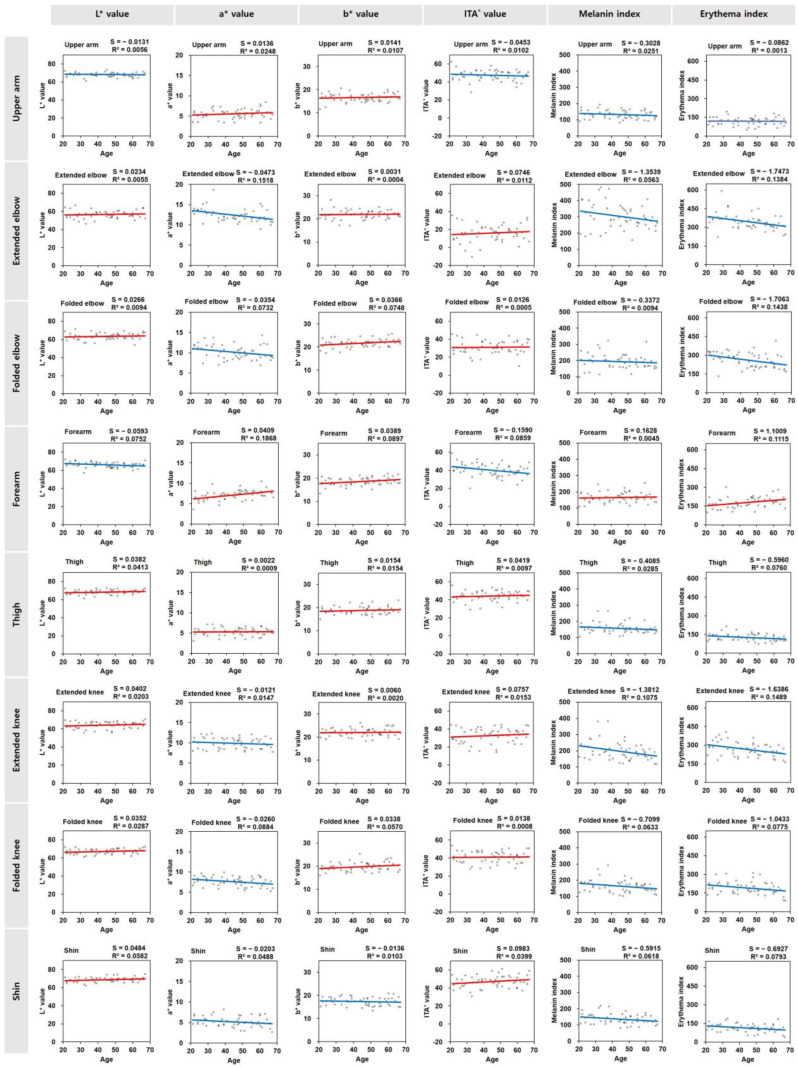
Scatter plots of the skin color parameter values in different skin sites versus the ages of the subjects (*N* = 53). A trend line is shown in each scatter plot, with numeric data of slope (S) and determination coefficient (R^2^) obtained from the simple linear regression analysis. Red and blue lines indicate upward and downward trends, respectively.

To determine the age-dependent changes in the skin color of each body part, scatter plots of the skin color parameter values of all subjects versus their age were drawn in Figure 4. The simple linear regression analysis provided the slope (S) and determination coefficient (R^2^) of the trend line in each scatter plot. 

The L* values exhibited age-dependent decreases in the forearm and upper arm skin sites, while the values of other skin sites tended to increase to different degrees. The a* values exhibited age-dependent increases in the forearm, upper arm, and thigh skin sites, while the values of other skin sites tended to decrease to different degrees. The b* values exhibited age-dependent increases to different degrees in most body skin sites except the shin skin site. The ITA° value exhibited age-dependent decreases in the forearm and upper arm skin sites, while the values of other skin sites tended to increase to different degrees. Both the melanin index and erythema index exhibited age-dependent increases in the forearm and decreases in other skin sites to different degrees. 

### 3.4. Correlation of Skin Color Parameter Values and the Ages of the Subjects

We examined whether the values of each skin color parameter are linearly associated with the ages of the subjects by performing Pearson correlation analysis. Pearson correlation coefficients (r) and *p* values are summarized in Table 4. The absolute values of r indicate that there is a very weak (0 < |r| < 0.2), weak (0.2 ≤ |r| < 0.4), moderate (0.4 ≤ |r| < 0.6), strong (0.6 ≤ |r| < 0.8), or very strong (0.8 ≤ |r| < 1) correlation between the two variables. The minus sign of the r value indicates a negative correlation. 

The data showed that the a* value of the forearm had a moderative correlation with age, and its b* value and erythema index had weak correlations with age, while its L* value and ITA° value had weak negative correlations with age. The a* value and erythema index of the extended elbow had weak negative correlations with age, and the b* value and erythema index of the folded elbow had weak negative correlations with age. The melanin index and erythema index of the extended knee had weak negative correlations with age, and the a* value and erythema index of the folded knee had weak negative correlations with age. The erythema indices of the thigh and shin had weak negative correlations with age.

## 4. Discussion

The analysis of skin color in Korean women of different age groups in the current study supports the hypothesis that age-related skin color change varies by the body part; however, further verification is needed in male subjects, and other races with different skin colors is additionally needed. 

The skin colors of the elbows and knees were darker (low L* and ITA° values and a high melanin index), redder (high a* value and erythema index), and more yellow (a high b* value) compared to that of other body parts, probably because the former two body parts have bumpy and wrinkled skin appearance. In particular, in the extended position, the skin in the elbow and knee area is wrinkled and the skin color appears dark. In the folded position, the skin in these body parts is stretched and the skin color appears less dark. The skin color of the elbows and knees may have been affected by their intrinsic folding and unfolding movements. In addition, the reason why the elbows had a darker and redder skin color than the knees may be explained by the differences in movement types, degrees, and frequencies between these two body parts [31]. 

Although the skin color of the forearm was lighter than that of the elbow and knee, it was darker and redder than that of the upper arm, thigh, and shin. This may be because the skin of the forearm is more exposed to various external factors, such as sunlight, ultraviolet rays, pollution, and friction than other body parts [27,28,37]. 

The skin color of various body parts exhibited different degrees of age-dependent changes. In particular, the skin of the forearm tended to darken (decreases in the L* and ITA° values and an increase in the melanin index) and become redder (increases in the a* value and the erythema index) and more yellow (an increase in the b* value) with age most significantly, followed by the upper arm skin, probably because these skin areas are easily exposed to various external factors during indoor and outdoor activities. 

On the other hand, in the case of the elbows and knees, the brightness and yellowness of the skin slightly increased, and the redness significantly decreased as the subject’s age increased, which was the opposite trend to the forearm. The cause of these changes is currently unclear, but it may be because the skin of the elbows and knees gradually becomes thicker, and the complexion becomes hidden or less visible. Alternatively, it may be due to decreased subcutaneous peripheral blood circulation in these areas due to intrinsic aging. Additional research is needed to examine these possibilities. 

In this study, when comparing skin color between different body parts or analyzing age-related changes in the skin color of each body part, it was found that the change in the a* value was greater than the change in the L* value, and the melanin index and erythema index differed or changed to similar degrees. This suggests that differences and changes in skin color may have been caused by a variety of factors, including the content of black melanin and other pigments, inflammatory responses, and blood circulation [35].

Because arms and legs are more exposed in the summer, the desire to improve skin tones in these areas can increase. Thus, it may be necessary to take care of the skin on your arms and legs in addition to your face, neck, and hands. This study showed that the skin of the elbows and knees, which are involved in and affected by intrinsic mechanical movements [38], took on a darker and redder color. Topically applying a softener to the elbows and knees may help alleviate the effects of physical movement or friction on skin color, texture, and wrinkles. 

This study also showed that the skin of the forearm and upper arm changed to a darker and redder color with age. Topical application of cosmeceutical products to the forearms and upper arms may help prevent skin pigmentation and/or inflammation caused by various external factors. In particular, cosmeceuticals with UV absorption, anti-melanogenic, and anti-inflammatory activities are thought to help alleviate skin pigmentation symptoms in different body parts [13,19,21,39].

The subjects in this study are evenly distributed across various age groups, making it suitable for analyzing body skin color changes with age. On the other hand, the number and distribution of subjects are insufficient to analyze the correlation of body skin color versus different occupations or lifestyles. Therefore, extensive follow-up research is necessary to address this issue.

From a dermatological perspective, it is also necessary to pay attention to the decrease in the erythema index with age in several body parts that are less exposed to the outside. If this change is due to a decrease in peripheral blood flow in the subcutaneous tissue, it will provide a clue to understanding the pathology of skin barrier loss and dryness associated with intrinsic skin aging [40,41,42].

## 5. Conclusions

In conclusion, by analyzing the skin color of various body parts, we observed that the skin colors of the elbow and knee were darker than other body parts and that the skin color of the forearm and upper arm became darker as people aged. Thus, it is suggested that the areas of the elbow, knee, forearm, and upper arm should be new skincare targets to alleviate the darkening of skin color caused by the performance of intrinsic mechanical function and/or exposure to external factors, such as sunlight, ultraviolet rays, air pollution, and physical friction. Since redness has decreased with age in several body parts other than the forearm and upper arm, research is needed to determine whether this change is related to a decline in blood circulation or other physiological or pathological changes.

## Figures and Tables

**Table 1:** Age information of human subjects (*N* = 53).

Age Groups	N	Mean	SD	Minimum	Maximum
20s	11	25.36	2.91	21	29
30s	10	36.20	2.70	32	39
40s	12	46.42	2.35	41	49
50s	10	54.30	2.95	51	59
60s	10	62.90	2.73	60	67
Total	53	44.72	13.49	21	67

**Table 2:** Occupation and life-related information of human subjects (*N* = 53).

Item	Classification	All Subjects		20s Subjects		30s Subjects		40s Subjects		50s Subjects		60s Subjects	
		*N*	%	*N*	%	*N*	%	*N*	%	*N*	%	*N*	%
Occupation	Workers	14	26	5	45	6	60	1	8	1	10	1	10
	Student	4	8	4	36	0	0	0	0	0	0	0	0
	None	2	4	2	18	0	0	0	0	0	0	0	0
	Housewife	33	62	0	0	4	40	11	92	9	90	9	90
Workplace	Indoor	45	85	11	100	9	90	9	75	8	80	8	80
	Outdoor	0	0	0	0	0	0	0	0	0	0	0	0
	Both	8	15	0	0	1	10	3	25	2	20	2	20
Average sleep duration	Less than 5 h	2	4	1	9	0	0	0	0	0	0	1	10
	5 to 8 h	47	89	7	64	10	100	12	100	9	90	9	90
	8 h or longer	4	8	3	27	0	0	0	0	1	10	0	0
Exposure to sunlight or UV rays	Less than 1 h	28	53	9	82	3	30	7	58	4	40	5	50
	1 to 3 h	24	45	2	18	7	70	4	33	6	60	5	50
	3 h or longer	1	2	0	0	0	0	1	8	0	0	0	0
Exposure to repeated friction	Yes	5	9	0	0	0	0	4	33	0	0	1	10
	No	47	89	10	91	10	100	8	67	10	100	9	90
	History	1	2	1	9	0	0	0	0	0	0	0	0
Smoking cigarettes	None	53	100	11	100	10	100	12	100	10	100	10	100
	Less than 10	0	0	0	0	0	0	0	0	0	0	0	0
	10 or more	0	0	0	0	0	0	0	0	0	0	0	0
Dominant hand	Left	5	9	1	9	0	0	0	0	1	10	3	30
	Right	31	58	7	64	6	60	10	83	6	60	2	20
	Both	17	32	3	27	4	40	2	17	3	30	5	50

**Table 3:** Skin characteristics of human subjects (*N* = 53).

Item	Classification		All Subjects		20s Subjects		30s Subjects		40s Subjects		50s Subjects		60s Subjects	
			*N*	%	*N*	%	*N*	%	*N*	%	*N*	%	*N*	%
Facial skin types	Dry skin		24	45	1	9	1	10	9	75	4	40	9	90
	Neutral skin		14	26	4	36	4	0	1	8	5	50	0	0
	Oily skin		1	2	0	0	1	70	0	0	0	0	0	0
	Dry-oily skin		13	25	5	45	4	20	2	17	1	10	1	10
	Problematic		1	2	1	9	0	0	0	0	0	0	0	0
Body skin moisture	Arm	Moist	1	2	0	0	0	0	0	0	0	0	1	10
		Normal	25	47	8	73	5	50	3	25	4	40	5	50
		Dry	27	51	3	27	5	50	9	75	6	60	4	40
	Leg	Moist	0	0	0	0	0	0	0	0	0	0	0	0
		Normal	14	26	6	55	3	30	2	17	1	10	2	20
		Dry	39	74	5	45	7	70	10	83	9	90	8	80
Body skin oil	Arm	Excessive	1	2	1	9	0	0	0	0	0	0	0	0
		Normal	32	60	6	55	7	70	5	42	7	70	7	70
		Insufficient	20	38	4	36	3	30	7	58	3	30	3	30
	Leg	Excessive	1	2	1	9	0	0	0	0	0	0	0	0
		Normal	26	49	5	45	7	70	6	50	3	30	5	50
		Insufficient	26	49	5	45	3	30	6	50	7	70	5	50
Body skin texture	Arm	Soft	9	17	2	18	2	20	3	25	0	0	3	30
		Average	38	72	9	82	7	70	7	58	9	90	6	60
		Rough	6	11	0	0	1	10	2	17	1	10	1	10
	Leg	Soft	6	11	2	18	2	20	0	0	0	0	2	20
		Average	36	68	7	64	6	60	10	83	8	80	5	50
		Rough	11	21	2	18	2	20	2	17	2	20	3	30
Body skin thickness	Arm	Thin	16	30	3	27	4	40	3	25	2	20	4	40
		Average	32	60	6	55	5	50	7	58	8	80	6	60
		Thick	5	9	2	18	1	10	2	17	0	0	0	0
	Leg	Thin	14	26	3	27	2	20	3	25	2	20	4	40
		Average	32	60	6	55	6	60	7	58	8	80	5	50
		Thick	7	13	2	18	2	20	2	17	0	0	1	10
Body skin pigmentation	Arm	High	11	21	2	18	2	20	1	8	3	30	3	30
		Normal	36	68	8	73	6	60	9	75	6	60	7	70
		Low	6	11	1	9	2	20	2	17	1	10	0	0
	Leg	High	9	17	2	18	1	10	1	8	2	20	3	30
		Normal	35	66	8	73	5	50	10	83	6	60	6	60
		Low	9	17	1	9	4	40	1	8	2	20	1	10

**Table 4:** Linear correlation analysis of the values of each skin color parameter of various body parts versus the ages of the subjects (*N* = 53). Pearson correlation coefficients (r) and *p* values are shown. A moderate correlation (r > 0.4), weak correlations (0.2 ≤ r < 0.4), and negative weak correlations (−0.4 < r ≤ −0.2) with a statistical significance (*p* < 0.05) are indicated with bold letters.

Skin Sites	The L* Value		The a* Value		The b* Value		The ITA° Value		Melanin Index		Erythema Index	
	r	*p*	r	*p*	r	*p*	r	*p*	r	*p*	r	*p*
Upper arm	−0.075	0.596	0.158	0.258	0.103	0.462	−0.101	0.473	−0.158	0.257	−0.036	0.796
											
Extended elbow	0.074	0.597	**−0.390**	**0.004**	0.021	0.881	0.106	0.451	−0.237	0.087	**−0.372**	**0.006**
			**(weak, negative)**								**(weak, negative)**	
Folded elbow	0.097	0.488	−0.271	0.050	**0.274**	**0.047**	0.023	0.870	−0.097	0.489	**−0.379**	**0.005**
					**(weak)**						**(weak, negative)**	
Forearm	**−0.274**	**0.047**	**0.432**	**0.001**	**0.299**	**0.029**	**−0.293**	**0.033**	0.067	0.632	**0.334**	**0.015**
	**(weak, negative)**		**(moderate)**		**(weak)**		**(weak, negative)**				**(weak)**	
Thigh	0.203	0.144	0.030	0.830	0.124	0.376	0.099	0.482	−0.169	0.226	**−0.276**	**0.046**
											**(weak, negative)**	
Extended knee	0.142	0.309	−0.122	0.385	0.044	0.754	0.124	0.378	**−0.328**	**0.017**	**−0.386**	**0.004**
									**(weak, negative)**		**(weak, negative)**	
Folded Knee	0.169	0.226	**−0.297**	**0.031**	0.239	0.085	0.028	0.840	−0.252	0.069	**−0.278**	**0.044**
			**(weak, negative)**								**(weak, negative)**	
Shin	0.241	0.082	−0.221	0.111	-0.102	0.470	0.200	0.152	−0.249	0.073	**−0.282**	**0.041**
											**(weak, negative)**	

## Data Availability

Data may be available from Ji Hwoon Baek upon request due to ethical reasons.
